# {2,6-Bis[1-(phenyl­imino)eth­yl]pyridine-κ^3^ *N*,*N*′,*N*′′}dichloridocobalt(II)

**DOI:** 10.1107/S1600536808013007

**Published:** 2008-05-07

**Authors:** Xiao-Gang Li, Di-Chang Zhong, Ren He, Hui-Rui Guo

**Affiliations:** a Dalian University of Technology, State Key Laboratory of Fine Chemicals, Dalian 116012, People’s Republic of China; b Institute of Coordination Catalysis, Yichun University, Yichun, Jiangxi 336000, People’s Republic of China

## Abstract

In the title complex, [CoCl_2_(C_21_H_19_N_3_)], the Co^II^ atom is coordinated by one pyridine and two imine N atoms and by two chloride anions in a distorted trigonal bipyramidal geometry. The structure exhibits a pseudo-mirror plane through the metal atom, two chloride anions and the pyridine ring. In the crystal structure, the complexes are connected via inter­molecular C—H⋯Cl hydrogen bonding.

## Related literature

For related literature on crystal structures of metal complexes of Schiff bases, see: Reardon *et al.* (2002 ▶); Pradhan *et al.* (2003 ▶); Gibson *et al.* (2001 ▶); Trivedi *et al.* (2007 ▶); Mentes *et al.* (2001 ▶); Esteruelas *et al.* (2003 ▶).
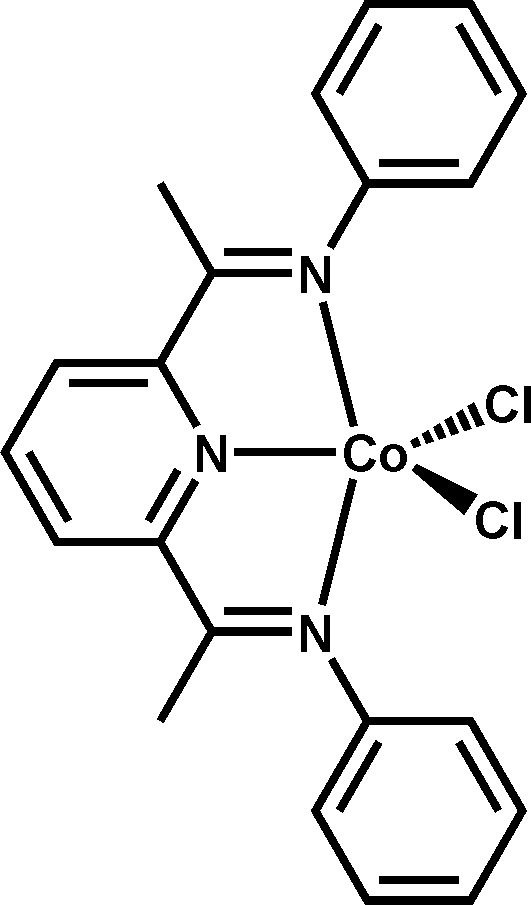


## Experimental

### 

#### Crystal data

[CoCl_2_(C_21_H_19_N_3_)]
*M* *_r_* = 443.22
Monoclinic, 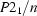

*a* = 10.4580 (3) Å
*b* = 15.2575 (4) Å
*c* = 13.1339 (3) Å
β = 95.825 (10)°
*V* = 2084.86 (9) Å^3^
*Z* = 4
Mo *K*α radiation
μ = 1.09 mm^−1^
*T* = 273 (2) K
0.36 × 0.30 × 0.28 mm

#### Data collection

Bruker SMART CCD area-detector diffractometer
Absorption correction: none
11050 measured reflections
3665 independent reflections
2594 reflections with *I* > 2σ(*I*)
*R*_int_ = 0.046

#### Refinement

*R*[*F* ^2^ > 2σ(*F* ^2^)] = 0.036
*wR*(*F* ^2^) = 0.075
*S* = 1.01
3665 reflections
246 parameters
H-atom parameters constrained
Δρ_max_ = 0.28 e Å^−3^
Δρ_min_ = −0.26 e Å^−3^

### 

Data collection: *SMART* (Bruker, 2004 ▶); cell refinement: *SAINT* (Bruker, 2004 ▶); data reduction: *SAINT*; program(s) used to solve structure: *SHELXS97* (Sheldrick, 2008 ▶); program(s) used to refine structure: *SHELXL97* (Sheldrick, 2008 ▶); molecular graphics: *SHELXTL* (Sheldrick, 2008 ▶); software used to prepare material for publication: *SHELXTL*.

## Supplementary Material

Crystal structure: contains datablocks global, I. DOI: 10.1107/S1600536808013007/nc2100sup1.cif

Structure factors: contains datablocks I. DOI: 10.1107/S1600536808013007/nc2100Isup2.hkl

Additional supplementary materials:  crystallographic information; 3D view; checkCIF report

## Figures and Tables

**Table:** 

Co1—N23	2.027 (2)
Co1—N24	2.208 (2)
Co1—N22	2.223 (2)
Co1—Cl2	2.2572 (8)
Co1—Cl1	2.2638 (8)

**Table:** 

N23—Co1—N24	75.36 (8)
N23—Co1—N22	75.38 (8)
N24—Co1—N22	150.74 (9)
N23—Co1—Cl2	119.07 (6)
N24—Co1—Cl2	96.11 (6)
N22—Co1—Cl2	98.36 (6)
N23—Co1—Cl1	123.81 (6)
N24—Co1—Cl1	96.23 (6)
N22—Co1—Cl1	99.58 (6)
Cl2—Co1—Cl1	117.03 (3)

**Table 2:** Hydrogen-bond geometry (Å, °)

*D*—H⋯*A*	*D*—H	H⋯*A*	*D*⋯*A*	*D*—H⋯*A*
C2—H2⋯Cl2^i^	0.93	2.67	3.545 (3)	156
C7—H7*A*⋯Cl1^ii^	0.96	2.76	3.663 (3)	158
C18—H18⋯Cl2^iii^	0.93	2.83	3.714 (3)	160

Symmetry codes: (i) 
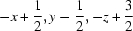
; (ii) 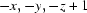; (iii) 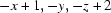.

**Fig. 1.**
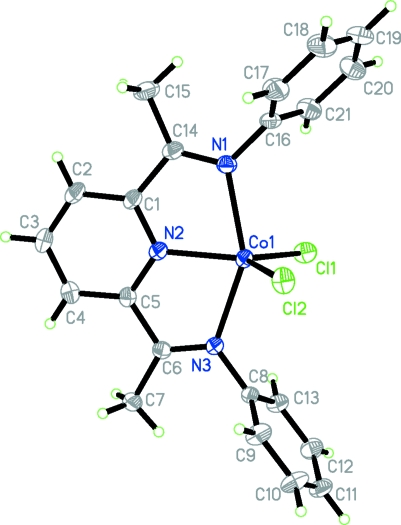
Crystal structure of the title compound with 30% probability displacement ellipsoids and the atom-labeling scheme.

**Fig. 2.**
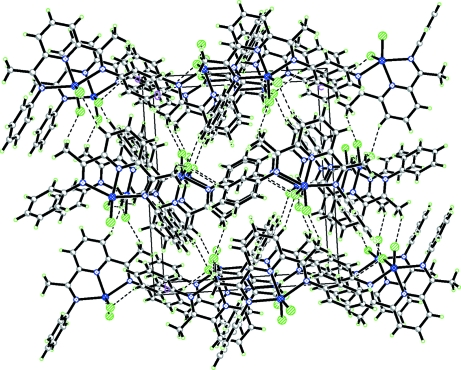
Crystal structure of the title compound with C—H···Cl hydrogen bonding shown as dashed lines.

## supplementary crystallographic information

### Comment

Recently, numerous crystal structures of metal-organic complexes with Schiff base ligands derived from 2,6-diacetylpyridine have been reported (Reardon *et al.*, 2002; Esteruelas *et al.*, 2003; Pradhan *et al.*, 2003; Gibson *et al.*, 2002; Trivedi *et al.*, 2007) in last several years. In our ongoing investigations on this topic we report here the crystal structure of the title compound.

In the crystal stucture of the title compound the Co^II^ atom is coordinated by three N atoms from the Schiff base ligand and two Cl atoms within a distorted trigonal-bipyramid geometry (Table 1 and Fig. 1). The pyridyl N atom and the two Cl atoms are located in the equatorial plane and the apical position are occupied by the two imino N atoms.

The molecules are connected by intermolecular nonclassical C—H···Cl hydrogen bonding between the aromatic and methyl H atoms and the Cl atoms (Table 2 and Fig 2).

### Experimental

The ligand Plep (2,6-bis[(1-phenylimino)ethyl]pyridine) was prepared in high yield from condensation of two equivalents of aniline with one equivalent of 2,6-diacetylpyridine in methanol according to the literature (Mentes *et al.*, 2001). The title compound was synthesized as follows: To a solution of Plep (1 mmol) in 10 mL methanol, a solution of CoCl_2_.6H_2_O (1 mmol) in 10 mL methanol was added dropwise at 333 K. After stirring for half an hour, the mixture was allowed to cool to room temperature and filtered off. On slow evaporation of the solvent from the filtrate at room temperature, red well shaped single crystals of the title compound were obtained in one week.

### Refinement

All H atoms were placed in geometrically idealized positions (,ethyl H atoms allowed to rotate but not to tip) and constrained to ride on their parent atoms, with C—H distances of 0.93 Å (0.96 Å for methyl H atoms) *U*_iso_(H) = 1.2*U*_eq_(C) (1.5 for methyl H atoms).

Data collection: *SMART* (Bruker, 2004); cell refinement: *SAINT* (Bruker, 2004); data reduction: *SAINT*; program(*s*) used to solve structure: *SHELXS97* (Sheldrick, 1997*a*); program(*s*) used to refine structure: *SHELXL97* (Sheldrick, 1997*a*); molecular graphics: *SHELXTL* (Sheldrick, 1997*b*); software used to prepare material for publication: *SHELXTL*.

### Crystal data

**Table:**

[CoCl_2_(C_21_H_19_N_3_)]	*F*_000_ = 908
*M**_r_* = 443.22	*D*_x_ = 1.412 Mg m^−^^3^
Monoclinic, *P*2_1_/*n*	Mo *K*α radiation λ = 0.71073 Å
Hall symbol: -P 2yn	Cell parameters from 1843 reflections
*a* = 10.4580 (3) Å	θ = 2.1–25.0º
*b* = 15.2575 (4) Å	µ = 1.09 mm^−^^1^
*c* = 13.1339 (3) Å	*T* = 273 (2) K
β = 95.8250 (10)º	Block, red
*V* = 2084.86 (9) Å^3^	0.36 × 0.30 × 0.28 mm
*Z* = 4	

### Data collection

**Table:**

Bruker SMART CCD area-detector diffractometer	2594 reflections with *I* > 2σ(*I*)
Radiation source: fine-focus sealed tube	*R*_int_ = 0.046
Monochromator: graphite	θ_max_ = 25.0º
*T* = 273(2) K	θ_min_ = 2.1º
φ and ω scans	*h* = −10→12
Absorption correction: none	*k* = −15→18
11050 measured reflections	*l* = −15→15
3665 independent reflections	

### Refinement

**Table:**

Refinement on *F*^2^	Secondary atom site location: difference Fourier map
Least-squares matrix: full	Hydrogen site location: inferred from neighbouring sites
*R*[*F*^2^ > 2σ(*F*^2^)] = 0.036	H-atom parameters constrained
*wR*(*F*^2^) = 0.075	*w* = 1/[σ^2^(*F*_o_^2^) + (0.0286*P*)^2^ + 0.0512*P*] where *P* = (*F*_o_^2^ + 2*F*_c_^2^)/3
*S* = 1.01	(Δ/σ)_max_ = 0.003
3665 reflections	Δρ_max_ = 0.28 e Å^−^^3^
246 parameters	Δρ_min_ = −0.26 e Å^−^^3^
Primary atom site location: structure-invariant direct methods	Extinction correction: none

### Special details

**Table:**

Geometry. All esds (except the esd in the dihedral angle between two l.s. planes) are estimated using the full covariance matrix. The cell esds are taken into account individually in the estimation of esds in distances, angles and torsion angles; correlations between esds in cell parameters are only used when they are defined by crystal symmetry. An approximate (isotropic) treatment of cell esds is used for estimating esds involving l.s. planes.
Refinement. Refinement of F^2^ against ALL reflections. The weighted R-factor wR and goodness of fit S are based on F^2^, conventional R-factors R are based on F, with F set to zero for negative F^2^. The threshold expression of F^2^ > 2sigma(F^2^) is used only for calculating R-factors(gt) etc. and is not relevant to the choice of reflections for refinement. R-factors based on F^2^ are statistically about twice as large as those based on F, and R- factors based on ALL data will be even larger.

### Fractional atomic coordinates and isotropic or equivalent isotropic displacement parameters (Å^2^)

**Table:**

	*x*	*y*	*z*	*U*_iso_*/*U*_eq_	
Co1	0.24632 (4)	0.03924 (2)	0.67087 (3)	0.03166 (12)	
Cl1	0.09612 (7)	0.14576 (5)	0.67423 (5)	0.0441 (2)	
Cl2	0.45297 (7)	0.07827 (5)	0.71366 (6)	0.0448 (2)	
N24	0.2555 (2)	0.04147 (15)	0.50369 (15)	0.0316 (5)	
N22	0.2133 (2)	−0.03183 (16)	0.81359 (15)	0.0343 (6)	
C6	0.2297 (2)	−0.03113 (19)	0.45798 (19)	0.0301 (6)	
N23	0.20616 (19)	−0.08547 (15)	0.62497 (16)	0.0316 (5)	
C14	0.1919 (3)	−0.1139 (2)	0.8020 (2)	0.0353 (7)	
C3	0.1514 (3)	−0.2543 (2)	0.5634 (2)	0.0436 (8)	
H3	0.1323	−0.3115	0.5425	0.052*	
C5	0.2025 (2)	−0.10652 (18)	0.5251 (2)	0.0308 (6)	
C1	0.1845 (2)	−0.14705 (19)	0.6945 (2)	0.0331 (7)	
C2	0.1571 (3)	−0.23215 (19)	0.6658 (2)	0.0412 (7)	
H2	0.1426	−0.2742	0.7146	0.049*	
C4	0.1742 (3)	−0.19112 (19)	0.4923 (2)	0.0387 (7)	
H4	0.1705	−0.2052	0.4231	0.046*	
C7	0.2247 (3)	−0.0470 (2)	0.34516 (19)	0.0423 (8)	
H7A	0.1407	−0.0677	0.3200	0.063*	
H7B	0.2878	−0.0901	0.3320	0.063*	
H7C	0.2424	0.0067	0.3112	0.063*	
C16	0.2226 (3)	0.0074 (2)	0.9132 (2)	0.0370 (7)	
C21	0.1319 (3)	0.0674 (2)	0.9352 (2)	0.0496 (9)	
H21	0.0644	0.0812	0.8862	0.059*	
C8	0.2866 (3)	0.11637 (18)	0.44589 (18)	0.0310 (7)	
C9	0.4113 (3)	0.1313 (2)	0.4257 (2)	0.0484 (8)	
H9	0.4758	0.0919	0.4487	0.058*	
C12	0.2231 (3)	0.2498 (2)	0.3586 (2)	0.0487 (8)	
H12	0.1595	0.2900	0.3362	0.058*	
C13	0.1924 (3)	0.1760 (2)	0.41291 (19)	0.0406 (8)	
H13	0.1081	0.1668	0.4271	0.049*	
C15	0.1728 (3)	−0.1794 (2)	0.8837 (2)	0.0557 (9)	
H15A	0.2453	−0.2183	0.8919	0.084*	
H15B	0.0961	−0.2126	0.8645	0.084*	
H15C	0.1648	−0.1495	0.9470	0.084*	
C10	0.4405 (3)	0.2045 (2)	0.3714 (2)	0.0552 (9)	
H10	0.5248	0.2140	0.3572	0.066*	
C20	0.1406 (3)	0.1075 (2)	1.0304 (2)	0.0615 (10)	
H20	0.0791	0.1485	1.0448	0.074*	
C18	0.3299 (4)	0.0281 (2)	1.0814 (2)	0.0651 (11)	
H18	0.3971	0.0145	1.1307	0.078*	
C11	0.3473 (3)	0.2633 (2)	0.3383 (2)	0.0505 (9)	
H11	0.3681	0.3126	0.3018	0.061*	
C17	0.3232 (3)	−0.0119 (2)	0.9863 (2)	0.0546 (9)	
H17	0.3862	−0.0516	0.9716	0.065*	
C19	0.2392 (4)	0.0873 (3)	1.1032 (2)	0.0630 (10)	
H19	0.2442	0.1138	1.1672	0.076*	

### Atomic displacement parameters (Å^2^)

**Table:**

	*U*^11^	*U*^22^	*U*^33^	*U*^12^	*U*^13^	*U*^23^
Co1	0.0368 (2)	0.0268 (2)	0.0312 (2)	−0.00071 (18)	0.00279 (16)	0.00155 (18)
Cl1	0.0371 (4)	0.0479 (5)	0.0480 (4)	0.0097 (4)	0.0080 (3)	0.0079 (4)
Cl2	0.0348 (4)	0.0449 (5)	0.0538 (5)	0.0047 (4)	0.0005 (3)	−0.0052 (4)
N24	0.0375 (14)	0.0279 (14)	0.0297 (12)	−0.0017 (11)	0.0045 (10)	0.0018 (11)
N22	0.0395 (14)	0.0349 (16)	0.0289 (13)	0.0041 (12)	0.0053 (10)	0.0033 (11)
C6	0.0260 (15)	0.0336 (18)	0.0310 (15)	0.0001 (13)	0.0042 (12)	0.0003 (14)
N23	0.0330 (13)	0.0288 (14)	0.0331 (13)	0.0000 (11)	0.0040 (10)	0.0038 (11)
C14	0.0356 (17)	0.0346 (19)	0.0364 (16)	0.0044 (14)	0.0070 (13)	0.0094 (14)
C3	0.0452 (19)	0.0274 (18)	0.057 (2)	−0.0019 (14)	0.0012 (16)	−0.0011 (16)
C5	0.0261 (15)	0.0296 (17)	0.0365 (16)	−0.0010 (13)	0.0017 (12)	−0.0026 (13)
C1	0.0316 (16)	0.0270 (18)	0.0409 (17)	0.0028 (13)	0.0054 (13)	0.0065 (14)
C2	0.0419 (19)	0.0287 (19)	0.0535 (19)	0.0042 (14)	0.0065 (15)	0.0129 (15)
C4	0.0373 (17)	0.037 (2)	0.0413 (17)	0.0034 (14)	0.0000 (14)	−0.0058 (15)
C7	0.052 (2)	0.042 (2)	0.0339 (16)	−0.0104 (15)	0.0106 (14)	−0.0066 (14)
C16	0.0446 (18)	0.0403 (19)	0.0274 (15)	−0.0006 (15)	0.0099 (14)	0.0070 (14)
C21	0.047 (2)	0.059 (2)	0.0435 (18)	0.0041 (17)	0.0103 (15)	−0.0010 (17)
C8	0.0398 (17)	0.0307 (17)	0.0223 (14)	−0.0041 (14)	0.0025 (12)	0.0012 (13)
C9	0.0406 (19)	0.048 (2)	0.057 (2)	0.0016 (16)	0.0094 (16)	0.0159 (17)
C12	0.062 (2)	0.042 (2)	0.0404 (18)	0.0053 (17)	−0.0005 (16)	0.0130 (16)
C13	0.0392 (18)	0.044 (2)	0.0393 (17)	0.0009 (15)	0.0049 (14)	0.0096 (15)
C15	0.071 (2)	0.051 (2)	0.0494 (19)	0.0050 (18)	0.0240 (17)	0.0184 (17)
C10	0.049 (2)	0.058 (3)	0.060 (2)	−0.0134 (18)	0.0118 (17)	0.0200 (19)
C20	0.068 (2)	0.067 (3)	0.054 (2)	0.003 (2)	0.0279 (19)	−0.008 (2)
C18	0.087 (3)	0.069 (3)	0.0357 (19)	0.008 (2)	−0.0104 (19)	0.0108 (18)
C11	0.071 (2)	0.045 (2)	0.0363 (17)	−0.0114 (19)	0.0070 (17)	0.0135 (16)
C17	0.069 (2)	0.053 (2)	0.0396 (19)	0.0147 (18)	−0.0019 (17)	0.0086 (17)
C19	0.095 (3)	0.066 (3)	0.0295 (18)	−0.008 (2)	0.0150 (19)	0.0039 (18)

### Geometric parameters (Å, °)

**Table:**

Co1—N23	2.027 (2)	C16—C21	1.369 (4)
Co1—N24	2.208 (2)	C16—C17	1.382 (4)
Co1—N22	2.223 (2)	C21—C20	1.387 (4)
Co1—Cl2	2.2572 (8)	C21—H21	0.9300
Co1—Cl1	2.2638 (8)	C8—C9	1.376 (4)
N24—C6	1.276 (3)	C8—C13	1.378 (4)
N24—C8	1.428 (3)	C9—C10	1.377 (4)
N22—C14	1.278 (3)	C9—H9	0.9300
N22—C16	1.434 (3)	C12—C11	1.368 (4)
C6—C5	1.495 (4)	C12—C13	1.388 (4)
C6—C7	1.497 (3)	C12—H12	0.9300
N23—C1	1.345 (3)	C13—H13	0.9300
N23—C5	1.347 (3)	C15—H15A	0.9600
C14—C1	1.495 (4)	C15—H15B	0.9600
C14—C15	1.495 (4)	C15—H15C	0.9600
C3—C4	1.380 (4)	C10—C11	1.363 (4)
C3—C2	1.382 (4)	C10—H10	0.9300
C3—H3	0.9300	C20—C19	1.368 (4)
C5—C4	1.384 (4)	C20—H20	0.9300
C1—C2	1.374 (4)	C18—C19	1.361 (5)
C2—H2	0.9300	C18—C17	1.386 (4)
C4—H4	0.9300	C18—H18	0.9300
C7—H7A	0.9600	C11—H11	0.9300
C7—H7B	0.9600	C17—H17	0.9300
C7—H7C	0.9600	C19—H19	0.9300
			
N23—Co1—N24	75.36 (8)	H7A—C7—H7C	109.5
N23—Co1—N22	75.38 (8)	H7B—C7—H7C	109.5
N24—Co1—N22	150.74 (9)	C21—C16—C17	119.4 (3)
N23—Co1—Cl2	119.07 (6)	C21—C16—N22	119.2 (3)
N24—Co1—Cl2	96.11 (6)	C17—C16—N22	121.4 (3)
N22—Co1—Cl2	98.36 (6)	C16—C21—C20	120.1 (3)
N23—Co1—Cl1	123.81 (6)	C16—C21—H21	120.0
N24—Co1—Cl1	96.23 (6)	C20—C21—H21	120.0
N22—Co1—Cl1	99.58 (6)	C9—C8—C13	119.4 (3)
Cl2—Co1—Cl1	117.03 (3)	C9—C8—N24	120.4 (3)
C6—N24—C8	119.6 (2)	C13—C8—N24	120.1 (2)
C6—N24—Co1	115.23 (18)	C10—C9—C8	119.9 (3)
C8—N24—Co1	125.20 (17)	C10—C9—H9	120.0
C14—N22—C16	120.8 (2)	C8—C9—H9	120.0
C14—N22—Co1	114.70 (18)	C11—C12—C13	119.7 (3)
C16—N22—Co1	124.34 (18)	C11—C12—H12	120.1
N24—C6—C5	115.7 (2)	C13—C12—H12	120.1
N24—C6—C7	126.2 (3)	C8—C13—C12	120.2 (3)
C5—C6—C7	118.1 (2)	C8—C13—H13	119.9
C1—N23—C5	120.3 (2)	C12—C13—H13	119.9
C1—N23—Co1	119.86 (18)	C14—C15—H15A	109.5
C5—N23—Co1	119.87 (18)	C14—C15—H15B	109.5
N22—C14—C1	115.8 (2)	H15A—C15—H15B	109.5
N22—C14—C15	127.2 (3)	C14—C15—H15C	109.5
C1—C14—C15	117.0 (3)	H15A—C15—H15C	109.5
C4—C3—C2	119.6 (3)	H15B—C15—H15C	109.5
C4—C3—H3	120.2	C11—C10—C9	120.7 (3)
C2—C3—H3	120.2	C11—C10—H10	119.7
N23—C5—C4	120.7 (3)	C9—C10—H10	119.7
N23—C5—C6	113.7 (2)	C19—C20—C21	120.4 (3)
C4—C5—C6	125.5 (3)	C19—C20—H20	119.8
N23—C1—C2	121.2 (3)	C21—C20—H20	119.8
N23—C1—C14	114.1 (3)	C19—C18—C17	120.6 (3)
C2—C1—C14	124.6 (3)	C19—C18—H18	119.7
C1—C2—C3	119.0 (3)	C17—C18—H18	119.7
C1—C2—H2	120.5	C10—C11—C12	120.1 (3)
C3—C2—H2	120.5	C10—C11—H11	120.0
C3—C4—C5	119.1 (3)	C12—C11—H11	120.0
C3—C4—H4	120.4	C16—C17—C18	119.8 (3)
C5—C4—H4	120.4	C16—C17—H17	120.1
C6—C7—H7A	109.5	C18—C17—H17	120.1
C6—C7—H7B	109.5	C18—C19—C20	119.7 (3)
H7A—C7—H7B	109.5	C18—C19—H19	120.2
C6—C7—H7C	109.5	C20—C19—H19	120.2

### Hydrogen-bond geometry (Å, °)

**Table:**

*D*—H···*A*	*D*—H	H···*A*	*D*···*A*	*D*—H···*A*
C2—H2···Cl2^i^	0.93	2.67	3.545 (3)	156
C7—H7A···Cl1^ii^	0.96	2.76	3.663 (3)	158
C18—H18···Cl2^iii^	0.93	2.83	3.714 (3)	160

Symmetry codes: (i) −*x*+1/2, *y*−1/2, −*z*+3/2; (ii) −*x*, −*y*, −*z*+1; (iii) −*x*+1, −*y*, −*z*+2.
